# The Tumor-Bed Boost Irradiation in Early-Stage Breast Cancer 30 Years After the Landmark Trials: What Is New?

**DOI:** 10.3390/jpm16090473

**Published:** 2026-09-15

**Authors:** Ilaria Benevento, Angela Solazzo, Francesca Tortoreto, Genoveva Ionela Boboc, Barnaba Floreno, Luciana Rago, Barbara D’Andrea, Antonietta Montagna, Antonella Bianculli, Aldo Piscitelli, Fabrizio Sanna, Salvatrice Campoccia, Antonella Ciabattoni, Grazia Lazzari

**Affiliations:** 1 Radiation Oncology Unit, Centro di Riferimento Oncologico della Basilicata, 85028 Rionero in Vulture, Italy; ilaria.benevento@crob.it (I.B.); angela.solazzo@crob.it (A.S.); luciana.rago@crob.it (L.R.); barbara.dandrea@crob.it (B.D.); antonietta.montagna@crob.it (A.M.); 2 Radiation Oncology Unit, Fatebenefratelli Hospital, 00186 Rome, Italy; francesca.tortoreto@fbf-isola.it; 3 UOSD Radioterapia, Radiation Oncology Unit, Ospedale Santa Maria Goretti, 04100 Latina, Italy; gi.boboc@ausl.latina.it; 4 UOSD Radioterapia, Radiation Oncology Unit, Ospedale Abele Aiello, 91026 Mazara del Vallo, Italy; barnabafloreno84@gmail.com; 5 Physic Unit, IRCCS-CROB, Centro di Riferimento Oncologico della Basilicata, 85028 Rionero in Vulture, Italy; antonella.bianculli@crob.it (A.B.); aldo.piscitelli@crob.it (A.P.); 6 Medical Physics Unit, Centro di Riferimento Oncologico della Basilicata, 85028 Rionero in Vulture, Italy; fabrizio.sanna@aouss.it (F.S.); salvatrice.campoccia@aouss.it (S.C.); 7 Radiation Oncology Unit, Ospedale San Filippo Neri, 00186 Rome, Italy; antonella.ciabattoni@aslroma1.it

**Keywords:** breast cancer, whole-breast irradiation, hypofractionation, DCIS, personalized radiotherapy

## Abstract

Breast-conserving surgery followed by adjuvant whole-breast irradiation (WBI) remains the standard of care for patients with early-stage breast cancer. In this setting, irradiation of the tumor bed with an additional boost dose has historically been used to reduce the risk of ipsilateral breast tumor recurrence (IBTR). Major randomized trials established that boost irradiation improved local control, particularly in younger patients, but did not confer a consistent overall survival (OS) benefit and was associated with increased late toxicity, like fibrosis, and poorer cosmetic outcomes as well. More recent evidence, including systematic reviews and contemporary consensus recommendations, suggests that boost irradiation may be safely omitted in selected low-risk patients, especially when the estimated absolute reduction in 10-year local recurrence is less than 3%. At the same time, the routine use of boost irradiation has been challenged by modern systemic therapy treatments questioning if boost could be really omitted in this set. Further advances in radiation techniques are redefining the scheduling and timing of boost administration, shifting from a conventional fractionation to hypofractionation and from the sequential (SEB) to simultaneous delivery with simultaneous integrated boost (SIB). In light of increasingly minimally invasive surgery and a de-escalation RT policy, unresolved clinical issues still remain about the management of positive or close margins and the role of boost irradiation in ductal carcinoma in situ. This narrative review summarizes the biological rationale, historical trial evidence, and recent literature addressing these evolving scenarios. Current evidence supports a more individualized approach to boost prescription based on recurrence risk, patient age, tumor biology, margin status, treatment techniques (surgery and radiotherapy), and patient preference.

## 1. Introduction

The rationale for delivering a tumor-bed boost after breast-conserving surgery is derived from the observation that most ipsilateral breast tumor recurrences occur close to the site of the primary lesion [1,2]. These findings provided the pathological basis for increasing the radiation dose to the tumor bed after WBI. Subsequently, five randomized trials evaluated the advantages and disadvantages of tumor-bed boost irradiation. Among these, the European Organisation for Research and Treatment of Cancer (EORTC) trial and its long-term updates clearly demonstrated that a boost improved local control in terms of low IBTR risks across all age groups, with the greatest absolute benefit observed in patients aged ≤40 years. However, this benefit comes at the cost of increased late toxicity, particularly fibrosis and worse cosmetic outcomes [3]. Despite the improvement in local control, no OS benefit was observed, even after 20 years of follow-up. In fact, the 20-year overall survival was 59.7% in the boost group versus 61.1% in the no-boost group, with a hazard ratio (HR) of 1.05 (99% CI 0.92–1.19, *p* = 0.323) [4]. These findings were subsequently confirmed by a Cochrane systematic review by Kindts et al., which included 8325 women from the five historical randomized trials. The review showed that tumor-bed boost irradiation significantly reduced local recurrence, without improving disease-free survival (DFS) or OS, and was associated with worse panel-assessed cosmesis [5]. Another review by Kayali et al. also confirmed a local-control advantage, particularly in younger patients, but again found no survival benefit [6].

Recently, on the basis of this background, the 2024 Assisi Think Tank Consensus (2024-ATTC) proposed that boost irradiation might be omitted when the estimated absolute reduction in 10-year local recurrence was <3%, whereas shared decision-making was recommended when the expected benefit exceeded this threshold [7]. In the modern era, the role of boost irradiation is being re-examined in light of improved systemic therapies, hypofractionated WBI, and more precise radiotherapy delivery techniques. In addition, clinically relevant questions remain regarding positive or close margins, ductal carcinoma in situ (DCIS), and the optimal sequencing of boost delivery.

This narrative review summarizes the biological rationale, historical evidence, and contemporary literature on tumor-bed boost irradiation in early-stage breast cancer, with a focus on these unresolved and aforementioned topics.

## 2. Literature Review Strategy

Because the role of tumor-bed boost irradiation has been extensively investigated in historical randomized trials with their subsequent updates, this narrative review focuses primarily on clinically relevant contemporary developments. Specifically, the review addressed the role of boost irradiation in challenging scenarios, including boost omission, margin status, hypofractionated radiotherapy, treatment sequencing, and DCIS.

### Materials and Methods

A literature search was conducted in PubMed/MEDLINE, EMBASE, and Google Scholar for articles published from 2005 to June 2026. Search terms included combinations of the following keywords: “breast cancer boost”, “breast cancer boost chemotherapy”, “breast boost trials”, “positive margins boost”, “hypofractionated radiotherapy boost”, “FAST-Forward boost”, and “DCIS and boost”. Boolean operators (AND/OR) were used to optimize the search strategy. Reference lists of relevant papers were manually screened, and recent data presented at major international meetings, including EBCC, ASTRO, and ESTRO, were also reviewed.

Eligible publications included peer-reviewed original articles, clinical trials, and review articles focused on tumor-bed boost irradiation in early-stage breast cancer. Studies dealing exclusively with locally advanced disease, neoadjuvant therapy, or post-mastectomy radiotherapy were excluded. Relevant ongoing and completed clinical trials were identified through ClinicalTrials.gov and the ISRCTN registry. Only articles published in English were included. No AI tools were used.

## 3. Biological Rationale for Tumor-Bed Boost Irradiation

Multiple clinical and pathological studies have shown that ipsilateral breast tumor recurrences after breast-conserving surgery predominantly arise near the original tumor site. In the 1980s, Holland et al. showed, through the examination of mastectomy specimens, that 60% of patients had residual cancer cells within 2 cm of the primary tumor margin, whereas only 11% had cancer cells as far as 4 cm away [1,2]. This observation has provided the clinical basis for administering an additional dose of radiotherapy to the tumor bed through several pivotal clinical trials.

Beyond this spatial pattern of recurrence, translational research has also provided a biological explanation for the benefit of boost irradiation. After surgery, physiological wound-healing processes activate inflammatory pathways and the epithelial-to-mesenchymal transition of stem cells, both of which are involved in tissue repair. Residual tumor cells within the surgical cavity may exploit these signals to restore proliferative and invasive capacities, thereby contributing to local recurrence [8,9].

Nuyten et al. evaluated several tumor gene-expression profiles of tumor tissue: the 70-gene prognosis profile, the hypoxia-induced profile, and the wound-response signature. Notably, he found that the wound-response signature was the only profile significantly associated with local recurrence, demonstrating that an activated wound pattern was significantly associated with increased local failure rates compared to a non-activated pattern [10]. Radiotherapy to the tumor bed has been shown to abrogate this proliferation. Belletti et al. demonstrated that wound fluid collected from non-irradiated surgical cavities stimulated breast cancer cell proliferation, migration, and invasion in vitro, whereas this effect was abolished when wound fluid was obtained from patients treated with intraoperative radiotherapy [11].These findings support the biological plausibility that tumor-bed irradiation may suppress wound-induced tumor regrowth and reinforce the radiobiological rationale for boost irradiation as a means of reducing the likelihood of local relapse. It cannot be ruled out that this treatment may also exert an immune-mediated systemic effect, when considering the tumor bed as an immunogenic hub [12,13].

## 4. Evidence from Literature

### 4.1. Pivotal Randomized Trials

Between 1986 and 1998, five pivotal randomized trials evaluated the role of tumor-bed boost irradiation after WBI in more than 8000 women. Despite differences in technique, dose, and patient selection, these studies consistently showed that boost irradiation improved local control, although at the cost of increased late toxicity.

The Lyon trial randomized 1024 patients to receive either a 10 Gy electron boost in four fractions or no boost. At a median follow-up of 3.3 years, the boost reduced the 5-year local recurrence rate, although it increased grade 1–2 telangiectasia [14].

The Budapest trial enrolled 207 women with stage I–II breast cancer treated with breast-conserving surgery and WBI. Patients were randomized to receive either a boost or no further radiotherapy. The boost significantly reduced crude local recurrence, with similar efficacies for electrons and high-dose-rate brachytherapy. However, side effects were more frequent in the boost arm [15]. In the 30-year update, DFS was 45% with the boost versus 38.9% without it (*p* = 0.0806), with no significant differences in local recurrence-free survival or distant metastasis-free survival. However, at 30 years, cancer-specific survival was significantly improved in the boost group (62% vs. 51.5%; *p* = 0.0282), although no statistically significant benefit in OS was observed (33% with boost vs. 27.9% without boost; *p* = 0.1182), with patients aged ≤40 years deriving the greatest benefit [16].

The Australian trial randomized 688 patients to receive either 50 Gy in 25 fractions to the whole breast or a boost arm within a reduced whole-breast dose of 45 Gy in 25 fractions followed by a 16 Gy electron boost in eight fractions. At a median follow-up of 8.5 years, the local recurrence rate was 2% in the control arm versus 4.4% in the boost arm, suggesting that reducing the whole-breast dose abrogated the clinical benefit of the radiotherapy boost. Nevertheless, the 5-year cosmetic outcomes, evaluated via breast retraction assessment, suggest that reducing the whole-breast dose may offset any potential benefit of the boost itself [17].

The Nice trial randomized 664 patients to receive no boost (Group A) or a 10 Gy boost (Group B) after WBI with 50 Gy in 25 fractions delivered via a telecobalt unit. At a median follow-up of 6 years, no statistically significant difference was found in local recurrence rates between the two cohorts (6.8% in Group A vs. 4.3% in Group B) [14].

The EORTC trial remains the most influential study in this field. It randomized 5569 patients with macroscopically excised tumors to receive either a 16 Gy boost or no boost after whole-breast irradiation. Among them, 2657 patients entered the boost arm. As a result, the addition of a boost significantly improved local control across all age groups, with the greatest absolute reduction in local recurrence observed in the younger cohort, albeit at the expense of increased toxicity. At a 10-year median follow-up, the cumulative incidence of local recurrence was 6.2% in the boost group versus 10.2% in the no-boost group [3]. However, severe fibrosis was significantly higher in the boost arm (10-year rate of severe fibrosis: 4.4% vs. 1.6%). The absolute risk reduction for local recurrence at 10 years was most pronounced in patients aged ≤40 years (13.5% vs. 23.9%). No survival benefit from the tumor-bed boost was observed. At 20 years, these difference were confirmed: OS was 59.7% (99% CI: 56.3–63.0) in the boost group versus 61.1% (99% CI: 57.6–64.3) in the no-boost group (HR: 1.05; 99% CI: 0.92–1.19; *p* = 0.323) [4]. Overall, these landmark trials established that tumor-bed boost irradiation improved local control but did not consistently improve survival and increased the risk of late toxicity and poorer cosmetic outcomes.

### 4.2. Systematic Reviews and Meta-Analyses

The results of the randomized trials were synthesized in a Cochrane review by Kindts et al., which included 8315 women. This analysis confirmed that tumor-bed boost irradiation significantly improved local control (HR: 0.64; 95% CI: 0.55–0.75; *p* < 0.00001). In a subgroup analysis of women older than 40 years, local control remained significantly superior with the boost (HR: 0.65; 95% CI: 0.53–0.81; *p* < 0.0001). However, no differences were found in disease-free survival (DFS) or OS. Panel-assessed cosmetic outcomes were significantly worse in the boost group, whereas physician-reported cosmetic results did not differ significantly [5].

A more recent review by Kayali et al. evaluated seven studies and found that six reported a significant local-control benefit with boost irradiation, with hazard ratios ranging from 0.34 to 0.73. The largest absolute benefit was observed in younger patients. Again, no study demonstrated an improvement in overall survival, while late fibrosis remained more frequent in patients receiving a boost. The cumulative incidence of severe fibrosis was significantly higher with boost therapy across all age groups older than 40 years (*p* < 0.001) [6]. The 2024-ATTC has summarized this evidence, suggesting boost omission on the basis of these thresholds: if the estimated absolute reduction in local recurrence is 1.5% at 5 years and less than 3% at 10 years with boost or, conversely, without boost if this estimated parameter is 3% at 5 years and 6% at 10 years [7]. Thus, a shared decision-making process with the patient should be implemented if the boost is expected to reduce the local recurrence rate by more than 3% at 10 years.

These data support the current view that the clinical value of boost irradiation lies primarily in improving local control rather than survival and that its use should be individualized according to recurrence risk and tolerance for late toxicity.

## 5. Can the Boost Be Safely Omitted in the Era of Modern Systemic Therapy?

Modern systemic therapy has markedly reduced the baseline risk of local recurrence in early-stage breast cancer. Endocrine therapy, chemotherapy, and anti-HER2 therapy all contribute substantially to improved local control, raising the question of whether boost irradiation is still necessary in all patients with early breast cancer after breast conservative surgery and undergoing modern adjuvant systemic therapy. Among drugs, it is well acknowledged that Tamoxifen reduces the risk of local recurrence by approximately 50%, while aromatase inhibitors and extended endocrine therapy may further reduce this risk [18,19]. Chemotherapy independently lowers the likelihood of local relapse, while trastuzumab appears to provide a similar benefit in HER2-positive disease. In this regard, in the HERA trial, 1082 patients treated with breast-conserving therapy, adjuvant chemotherapy, and trastuzumab were analyzed according to whether they received a tumor-bed boost. At a median follow-up of 11 years, local recurrence occurred in 7% of patients receiving a boost and 9% of those who did not, a difference that was not statistically significant (*p* = 0.33) [20,21]. Notably, even patients younger than 40 years did not achieve a statistically significant reduction in local recurrence from the boost. Currently, no specific trials are available, and data are limited to real-world evidence like results from a large Dutch national registry presented at ESTRO 2026, which further supports this approach from the thresholds suggested by 2024 ATTC and in selected risk groups [7]. The local relapse risk factors analyzed included ages of ≤40 years, grade 3 tumors, a triple-negative subtype, and positive surgical margins. The LR risk was evaluated for patients treated both with and without a boost, as well as for specific subgroups defined by these risk factors. In this analysis including 34,504 patients treated between 2012 and 2016, the 8.3-year local recurrence (LR) rate remained very low both with and without a boost, including in clinically relevant subgroups and remained well below the established clinical thresholds of 6% without a boost and 3% with a boost, even after adjusting for an 80% completeness index. Specifically, in patients with 0–2 risk factors, the LR rate was less than 1% regardless of whether a boost was administered [22]. These data suggest that routine boost irradiation may represent overtreatment in selected low-risk patients treated in the context of modern systemic therapy. Therefore, at present, boost omission should be considered cautiously and within an individualized decision-making framework.

## 6. Positive and Close Surgical Margins

### 6.1. Unresectable Positive Margins

Margin status remains one of the most important determinants of local recurrence risk. There is broad agreement that positive margins, defined as tumor cells on ink, are associated with a two-fold increased risk of ipsilateral breast tumor recurrence [23]. Whether boost is effective is controversial. A meta-analysis conducted by Moran et al., which evaluated 18 studies assessing boost utilization in patients with positive margins, found that the risk of local failure remained significantly elevated (OR: 2.45; *p* < 0.001) [24]. The role of high-dose boost irradiation in this setting still remains uncertain. A subgroup analysis of the EORTC trial including 251 patients with positive margins suggested a numerically lower recurrence rate with a 26 Gy boost compared with a 10 Gy boost. The 10-year IBTR incidence was 17.5% in the 10 Gy cohort compared to 10.8% in the 26 Gy cohort (*p* > 0.1). However, this dose escalation did not translate into a survival advantage (HR: 0.97; 95% CI: 0.59–1.5; *p* > 0.1). Conversely, the rate of moderate-to-severe fibrosis increased significantly from 24% to 54.3% (*p* < 0.0001), demonstrating a clear dose-dependent toxicity profile [25].

In the Budapest trial, which enrolled 207 patients, positive margins were reported in 9.7% of the no-boost arm and 6.7% of the boost arm. In the subgroup receiving a high-dose boost delivered via high-dose-rate (HDR) brachytherapy, the 5-year local recurrence (LR) rate was reduced from 11.6% to 6.8% in patients with negative margins, and precipitously dropped from 50.8% to 8.3% in those with positive or close margins. In the recent 30-year update, the local recurrence-free survival (LRFS) rate among patients with positive or close margins was 77% with the boost versus 20.5% in the no-boost group, whereas no significant difference was detected in the negative-margin cohort. However, this clinical benefit was offset by a high incidence of grade 3–4 fat necrosis in the HDR brachytherapy group [15,16].

In a retrospective study by Lee et al. evaluating 550 patients, a subgroup of 55 individuals with positive margins received a high-dose boost of 15 Gy in five fractions following 50 Gy of whole-breast irradiation (WBI). After a median follow-up of 58 months, the crude LR rate was 7.3% in the positive-margin group compared to 2.4% in the negative-margin cohort. Although positive margins were associated with a higher rate of local recurrence across the entire cohort, this difference did not reach statistical significance (*p* = 0.062). Notably, young patients with positive margins experienced a significantly lower 5-year LRFS rate than those with negative margins (89.16% vs. 97.57%, respectively; *p* = 0.005). In contrast, no significant difference in the 5-year LRFS rate was observed between the positive and negative margins among patients aged ≥60 years (100.00% vs. 94.38%, respectively; *p* = 0.426) [26]. The Young Boost Trial further highlighted the cost–benefit ratio of dose escalation, showing that at the 10-year follow-up, the local relapse rate was 2.8% in the 26 Gy group versus 4.4% in the 16 Gy group, but this benefit was accompanied by substantially increased severe fibrosis and worsened cosmetic outcomes. In fact, the cumulative incidence of severe fibrosis nearly doubled, rising from 27% in the 16 Gy group to 48% in the 26 Gy group. This increase in late toxicity occurred independently of the radiation modality (electrons vs. photons), boost volume, or baseline cosmetic scores [27]. Taken together, the available evidence suggests that although dose escalation may improve local control in selected patients with unresectable positive margins, its clinical value remains uncertain and must be weighed carefully against increased late toxicity.

### 6.2. Close Margins

The role of boost irradiation in patients with close but negative margins remains controversial. Practice patterns vary internationally, reflecting the absence of a universal consensus as shown by an American survey [28]. In an Australian survey, 88% of radiation oncologists considered a margin of <2 mm as a relative indication for a boost. Interestingly, 35.2% felt that a margin of <2 mm was an absolute indication, 38.7% considered it a relative indication, and 26.1% did not view it as an indication at all [29]. Close margins, defined as a no-ink tumor margin width of ≤2 mm, seem to have no significant impact on the risk of ipsilateral breast tumor recurrence (IBTR), as demonstrated by several studies [30,31]. In the meta-analysis by Moran, analyzing 19 studies including 13,081 patients at a median follow-up of 8.7 years, the relationship between specific margin widths (1, 2, and 5 mm) and IBTR was not statistically significant. Furthermore, no statistical trend was found linking an increased negative-margin width to a decreased rate of IBTR (*p* trend = 0.58). These findings were confirmed even after adjusting for covariates such as the use of a radiation boost or re-excision [24].

In a systematic review by Shah, which included 38 eligible studies comprising 54,502 patients treated between 1968 and 2010 (median follow-up of 7.25 years), absolute local recurrence (LR) rates decreased over time across all margin width cohorts. The maximum differences between negative-margin groups were less than 1% for the most recent enrollment period. However, the relative rates of LR between different margin groups remained stable over time [32]. Similarly, the meta-analysis conducted in 2014 by Houssami, based on 33 studies (1506 LR events out of 28,162 patients), showed that the odds of LR were strongly associated with margin status [model 1: odds ratio (OR) 1.96 for positive/close vs. negative; model 2: OR 1.74 for close vs. negative, and 2.44 for positive vs. negative; (*p* < 0.001 for both models)]. Conversely, LR was not associated with margin distance [model 1: 0 mm vs. 1 mm (referent) vs. 2 mm vs. 5 mm (*p* = 0.12); model 2: 1 mm (referent) vs. 2 mm vs. 5 mm (*p* = 0.90)], after adjusting for the median follow-up time of the studies [33].

In the EORTC reanalysis by Vrieling, the local recurrence benefit of boost irradiation was similar regardless of whether the negative margin was ≤2 mm, 3–4 mm, or ≥5 mm. At an 18.2-year follow-up, patients with close margins of ≥5 mm, 3–4 mm, and ≤2 mm achieved an identical local recurrence rate of 10% across all groups. This indicated an equal reduction in local recurrence risk regardless of whether the margin was ≥5 mm, 3–4 mm, or ≤2 mm (*p* = 0.63) [34]. On the contrary, Cho et al. evaluated 297 patients treated between 2000 and 2012, where 80.8% had positive margins and 18.2% had close margins < 1 mm; they reported that a higher dose (>66 Gy EQD2) improved local control even in patients with margins < 1 mm [35]. Tailoring the boost dose to margin width did not seem to provide a clinical advantage. In a study by Livi, a dose-escalation strategy tailored to the surgical margin width was tested, administering 10 Gy, 16 Gy, or 20 Gy for margins of >5 mm, 2–5 mm, and <2 mm, respectively. After a median follow-up of 5.2 years, 41 local relapses (LR, 2%) were recorded. Regarding local recurrence-free survival (LRFS), age at diagnosis, nuclear grade, hormonal status, T-stage, adjuvant hormonal therapy, and adjuvant chemotherapy emerged as significant parameters (*p*-values from log-rank test < 0.05). However, the final margin status, which dictated the radiation boost dose, did not have a significant impact on LRFS (*p* = 0.46). LR rates were 2.3% for FMS < 2 mm, 2.6% for 2–5 mm FMS, and 1.8% for FMS > 5 mm. In the multivariate analysis, higher nuclear grade (*p* = 0.045), triple-negative subtype (*p* = 0.036), and higher T-stage (*p* = 0.02) were identified as the only independent predictors of LR occurrence [36].

Accordingly, the current evidence does not support routine dose escalation solely on the basis of a close, negative margin. Decisions regarding boost prescription should instead be based on the overall recurrence-risk profile.

## 7. How Should the Boost Be Delivered: Sequential or Simultaneous Integrated Boost?

The traditional approach to boost delivery has been a sequential external-beam boost after completion of whole-breast irradiation (WBI) with conventional fractionation. Historical trials typically used sequential doses of 10–16 Gy, usually delivered after WBI in 5–8 fractions of 50 Gy with 2D or 3D techniques or with moderate hypofractionation like the Lyon trial [14]. With the adoption of intensity-modulated radiotherapy and hypofractionated whole-breast irradiation, simultaneous integrated boost (SIB) has emerged as an attractive alternative to a sequential delivery because SIB reduces the overall treatment time and the number of hospital visits while maintaining dose intensification to the tumor bed. Firstly, starting in 2005, several SIB schedules were also tested within conventional fractionation, such as 28 fractions of 1.81 Gy to the breast + 0.49 Gy extra as the boost or 21 fractions of 2.17 Gy to the breast + 0.49 Gy as boost. The study reported an actuarial 5-year local-control rate of 98.9%, minimizing ipsilateral recurrences [37].

The IMRT-MC2 trial was the first phase III study to compare SIB and sequential boost using conventional fractionation and novel techniques like IMRT. In this study, WBI with 50.4 Gy/64.4 Gy SIB/28 fractions versus 50.4 Gy/28 fractions + a 16 Gy boost to 66.4 Gy delivered in 8 fractions were compared. With a median follow-up of 5.1 years, 2-year local control in the SIB arm was not inferior to that in the SEB arm (99.6% vs. 99.6%, *p* = 0.487). This study also demonstrated the non-inferiority of cosmetic outcomes after IMRT-SIB and 3D-conformal SEB radiotherapy at 6 months (9.1% vs. 9.1%) and 2 years (10.4% vs. 9.8%) [38].

### 7.1. Boost Irradiation in Moderate Hypofractionated Schedule

More recently, preliminary results from the non-inferiority RTOG 1005 study were reported. In this study, approximately 2500 high-risk stage I-II patients were randomized to the standard arm of conventional WBI—50 Gy in 25 fractions and 42.7 Gy in 16 fractions plus a sequential boost of 12 Gy in 6 fractions versus 14 Gy in 7 fractions (Arm I) versus hypofractionated-WBI 40 Gy in 15 fractions plus a concomitant boost of 8 Gy in 15 fractions of 0.53 Gy per day (Arm II). With a median follow-up of 7.3 years, the 5-year data confirmed non-inferiority between Arms A and B, with no difference in adverse events, cosmetic outcome, or survival, using both 3D and IMRT techniques 35. The 5-year recurrence rate was 2.0% in Arm I versus 1.9% in Arm II; at 7 years, the recurrence rate was 2.2% in Arm I versus 2.6% in Arm II, so the statistical criterion for non-inferiority was fully met (*p* = 0.039). Clinical cosmetic assessment 3 years after treatment showed “excellent or good” results in 86% of patients in Arm I versus 84% in Arm II (*p* = 0.61), confirming that a moderate but more intensive hypofractionation did not worsen the aesthetic impact [39].

Several moderately hypofractionated SIB schedules have shown promising efficacy and safety, using 15- or 16-fraction approaches. The most used schedule is the Franceschini regimen, which provides 40 Gy in 15 fractions to the whole breast and 48 Gy SIB. At a median follow-up of 6 years, the local recurrence risk was 1.1%, with an excellent or good cosmetic outcome in 99% of cases [40]. Another SIB regimen consists of 40 Gy in 16 fractions to the breast and a 48 Gy SIB; at 5 years of follow-up, this schedule resulted in a 1% local recurrence rate, with no toxicity in 64% of cases and a low incidence of grade 3 toxicity (telangiectasia and tissue edema) [41]. In the randomized controlled trial by Van Hulle et al., 150 patients were treated in the prone position and a SEB schedule in moderate hypofractionation of 40.05 Gy in 15 fractions was compared with a SIB of 46.8 Gy (negative margins) or 49.95 Gy (positive margins). The study confirmed the safety of SIB in reducing treatment time without additional risks, with 2-year data showing no grade 3 toxicity or severe cosmetic deterioration between the two arms [42].

Randomized studies, such as IMPORT HIGH and HYPOSIB, have demonstrated non-inferior local control with acceptable toxicity [43,44].

Updates from the IMPORT HIGH trial have been recently reported. This study involved 2601 patients and included a control arm with WBI 40.05 Gy and a sequential boost of 16 Gy (2 Gy × 8 fractions), plus two comparison arms: (A) 36 Gy in 15 fractions to the residual breast associated with concomitant partial breast irradiation (APBI) of 40 Gy to a partial volume and a concomitant boost SIB of 48 Gy to a volume including the tumor bed defined by clips as CTV + 15 mm and 10 mm as PTV and (B) a concomitant boost SIB of 53 Gy. As a non-inferiority study for an absolute IBTR difference of 3% at 5 years, the criterion was met in the 48 Gy arm but not in the 53 Gy arm, compared with the standard arm. With a median follow-up of 74 months, the 5-year local recurrence rate was equivalent (1.9% vs. 2% vs. 3.2%), but greater toxicity, such as breast induration, was observed in the 53 Gy arm [43].

The 10-year update presented at ESTRO 2026 showed a cumulative 10-year IBTR rate of 3.5% for the standard arm versus 3.7% in Arm A and 5.5% in Arm B, with an absolute difference of 0.4% in A and 2% in B relative to the standard arm. The absolute difference at 10 years for local recurrence was 0.1 in Arm A and 2.2 in Arm B, while the incidence of cancer-related events was 0.4 in Arm A versus 2.5 in Arm B. The absolute difference in OS was −0.5 in A versus 1.5 in B. Using VMAT and IMRT techniques, the adoption of a hypofractionated SIB of 48 Gy in 15 fractions appears feasible and safe. Long-term subanalyses based on PROMS questionnaires showed no statistically significant difference in the perception of adverse symptoms affecting the breast, arm, or shoulder up to 5 years across the various arms. Moreover, treatment acceptance was very high, likely related to the strong positive impact of reducing hospital visits, with treatment completed in only 3 weeks instead of the traditional 5–6 weeks [45].

Preliminary results from the randomized phase III HYPOSIB non-inferiority trial were presented at ASTRO 2024. Between 2015 and 2019, 2310 patients were randomized to receive either hypofractionated whole-breast irradiation with a simultaneous integrated boost (40 Gy/48 Gy in 15 fractions) or the same whole-breast schedule followed by a sequential boost of 16 Gy in eight fractions. After five years, the SIB strategy proved non-inferior with respect to local control (98.2% vs. 98.0%; *p* = 0.84), disease-free survival (92.0% vs. 92.2%; *p* = 0.58), and overall survival (98.2% vs. 97.9%; *p*= 0.48). The incidence of grade ≥ 2 skin toxicity or fibrosis was similar between groups (7.3% vs. 8.4%), as was the incidence of telangiectasia (1.5% in both arms) [44].

The Hi-RISE clinical study is ongoing; it is a phase III non-inferiority trial on the local control of hypofractionated radiotherapy with a simultaneous boost of 48 Gy in 15 fractions (HFRTsib) compared with conventional fractionation with a SEB of 10 Gy in 5 fractions. The study plans to enroll 2904 women with early breast cancer and may be decisive regarding the use of SIB in moderate hypofractionation [46].

Within the context of moderate hypofractionation but accelerated trials, a prospective non-inferiority trial, “NOVEMBER,” should also be cited. It enrolled 103 patients treated with 34.2 Gy/9 fractions with a SIB of 39.6 Gy/9. At a median follow-up of 51 months, no local recurrence was recorded and only one patient had regional recurrence in the axilla and distant recurrence in the brain. Toxicity was equivalent and low in both arms at 2 years. Cosmetic results at 24 months were 68% excellent/good and 32% fair/poor. No late toxicity of ≥grade 3 was recorded [47].

### 7.2. Boost Irradiation in Ultra-Hypofractionated Schedules

Following the adoption of five-fraction whole-breast irradiation, interest has expanded toward integrating boost delivery into ultra-hypofractionated schedules. Both sequential and simultaneous approaches are under investigation.

Initial FAST-Forward-related experiences suggested that a sequential boost of 10.4 Gy in two fractions was associated with excessive toxicity, leading to the abandonment of that schedule. At 36 months, OS and local recurrence-free survival were 96% (95% CI, 94–99) and 93% (95% CI, 90–97), respectively. The highest acute and late toxicity rates were observed in the 10.4 Gy group compared with the no-boost or 5.2 Gy boost groups (37.4%, 10.8%, and 12.2% [acute] and 22.7%, 8.6%, and 7.9% [late], respectively) [48].

This schedule, however, was later reintroduced in a similar phase II study called SHIFT, evaluating a SEB of 10.4 Gy/2 fractions MV for invasive and in situ breast carcinoma. At a median follow-up of 28.3 months, no local recurrence, distant metastasis, or death were recorded [49].

The FAST-Forward boost study is currently in the recruitment phase; it includes a control arm with SIB 48 Gy in 15 fractions versus 31 Gy and 30 Gy in 5 fractions. More than 4000 patients are expected to be enrolled in this non-inferiority study. The aim is to test the non-inferiority of the SIB delivered in only five fractions (one week) compared with the standard SIB over 3 weeks, as emerged from IMPORT High [43,50].

Another study awaiting results is the Indian HYPORT Study, in which patients are randomized to 40 Gy/15 fractions with an 8 Gy SIB versus 26 Gy/5 fractions with a 6 Gy SIB, respectively. Grade 3 acute dermatitis was reported in 3/271 patients, but not in the SIB arm. Interim toxicity analysis indicates a minimal incidence of severe acute toxicity, without differentiation according to SIB dose or schedule [51]. Another study awaiting results is MC1635, in which patients are randomized to 40 Gy/15 fractions with an 8 Gy SIB versus 26 Gy/5 fractions with a 4 Gy SIB, respectively [52].

A real-world observational study is ongoing using SIB 6 Gy versus SEB 7.6 Gy/3.8 × 2 fractions, within the Italian multicenter Perugia study on intensified hypofractionation with sequential or concomitant boost, in which the SIB arm refers to the Florence APBI study [53,54]. Analysis is a work in progress. At present, ultra-hypofractionated SIB appears feasible, but caution is warranted until robust long-term efficacy and cosmetic data become available.

## 8. Boost Irradiation in Ductal Carcinoma In Situ

Local recurrence remains the most common form of treatment failure after breast-conserving surgery for DCIS in the presence of inadequate surgical margins, while a margin of at least 2 mm is currently recommended by consensus guidelines [55,56].

Historically, evidence supporting routine boost irradiation in DCIS has been limited. However, more recent studies have clarified its potential role according to the risk-level of the disease. Non-low-risk DCIS was defined by the following characteristics: age < 50 years, symptomatic tumor at diagnosis, palpable mass, lesion size ≥ 15 mm, multifocal disease, high or intermediate nuclear grade, central necrosis, comedo histology, or a radial surgical margin < 10 mm. In the randomized BIG 3-07/TROG 07.01 trial, boost irradiation significantly improved recurrence-related outcomes at 10 years in patients with non-low-risk DCIS, although no overall survival benefit was observed. In this study, 1608 patients with non-low-risk DCIS were randomized to receive a boost versus no boost, combined with either conventional fractionation or hypofractionation (4-arm design). The 10-year recurrence-free survival (RFS) rate was 86% in the no-boost group compared to 93.2% in the boost cohort. Similarly, the 10-year freedom from disease recurrence rate was 79.4% without a boost versus 86.5% with a boost (HR, 0.67; 95% CI, 0.52–0.86; *p* = 0.002). Conversely, the estimated 10-year overall survival (OS) rates were 93.9% in the no-boost group and 95.7% in the boost group, demonstrating no statistically significant difference (HR, 0.73; 95% CI, 0.47–1.14; *p* = 0.17). The study also demonstrated that the hypofractionated radiotherapy arm is non-inferior to standard treatment. Indeed, in Random A (four arms), the estimated 10-year freedom-from-local-recurrence rates were 89.5% for conventional fractionation and 88.6% for the hypofractionated arm (HR, 1.07; 95% CI, 0.62–1.84; *p* = 0.82) [57].

Nevertheless, late toxicity remains a critical consideration in DCIS management. In the Australian-SWG study, which included patients with DCIS followed for a median duration of 6.6 years, the addition of a boost significantly reduced the local recurrence (LR) rate from 7.3% to 2.9% (*p* < 0.001), albeit at the expense of increased adverse effects [17]. More recently, findings from the French BONBIS trial—which randomized 2000 patients to a 16 Gy boost in eight fractions—revealed an increase in local toxicity, specifically regarding the risk of grade ≥ 2 breast subcutaneous fibrosis (BSCF), which rose from 2.8% (without boost) to 7.0% (with boost) (*p* < 0.001). Grade ≥ 2 BSCF was significantly correlated with the administration of a boost (OR = 2.6; 95% CI, 1.62–4.3) and a breast clinical target volume (CTV) of ≥500 cm^3^ (OR = 1.6; 95% CI, 1.01–2.51). Furthermore, quality of life analyses using the QLQ-C30 and QLQ-B23 instruments demonstrated a higher frequency of persistent breast symptoms over time (*p* = 0.001) alongside an early detriment in body image (HR = 1.19; 95% CI, 1.00–1.41) [58]. Therefore, in DCIS, the use of boost irradiation should be individualized, with particular consideration of recurrence risk, expected benefit, toxicity, and quality of life.

## 9. Target Delineation and Boost Volume Considerations

An additional issue of increasing relevance in contemporary practice is boost target delineation, particularly in light of recent advances in oncoplastic surgery with the use of flaps procedure reconstructions and the adoption of hypofractionation. In this context, the tumor bed is displaced from the original site with perforant flapping dorsal muscle reconstruction [59] and little information is available on tumor-bed boost irradiation and long-term outcomes as assessed by a systematic review [60]. The hot spots in hypofractionated RT become critical for the boost due to ‘double and triple trouble’ phenomena [61]. Historically, following wide local excision, the boost clinical target volume (CTV) was defined around the surgical scar using a 1–2 cm isotropic margin This scar-based approach to boost-CTV delineation is now considered inadequate because it may fail to encompass the actual tumor-bed clips in at least 70% of cases [62]. The interobserver variation in the boost-CTV delineations has been advocated based on preoperative CT images, resulting in a significant reduction in the boost-CTV volume [63].

Current practice favors the use of titanium clips or fiducial markers placed within the surgical cavity to improve tumor-bed localization, as implemented in the IMPORT protocols [64,65]. Greater consistency in boost-CTV delineation may be achieved by following the current consensus recommendation, which advocates defining the CTV based on a clip-delineated tumor bed with an individualized margin to create the breast planning target volume (PTV) according to the center’s protocols, ensuring that the final boost volume is proportional to the total breast volume, particularly in hypofractionated SIB techniques. As a limit to adopt, it has been suggested that the PTV boost should not exceed 20% of the total breast PTV [41]. This issue is particularly important because the volume of irradiated tissue contributes significantly to fibrosis, induration, and cosmetic outcomes.

## 10. Discussion

Three decades following the landmark randomized trials, the clinical role of tumor-bed boost irradiation continues to be debated within the context of contemporary radiotherapy challenges. Its role remains unquestionable as an effective strategy for improving local control after breast-conserving surgery. The largest absolute benefit is consistently observed in younger patients and in those over 50 years with a higher baseline risk of local recurrence. However, this local-control advantage has not translated into a clear and consistent overall survival benefit, and it is offset by increased rates of fibrosis, induration, and poorer cosmetic outcomes. Among all the available studies, its adoption is driven by risk factors. Young age, large tumor size, positive surgical margins, high histological grade, lymphovascular invasion, extensive intraductal component (EIC), triple-negative subtype, and poor response to systemic therapy in HER2-positive or triple-negative disease have been confirmed as relevant risk factors to determining elements in the decision-making process for boost administration as shown in Table 1 and Table 2 [7,66].

In the current era, the most important question is no longer whether boost irradiation works, but rather in whom it is still necessary, especially when effective long-term systemic therapy is provided [67]. Modern systemic therapy has substantially reduced local recurrence risk, and emerging real-world data suggest that boost omission may be safe in carefully selected low-risk patients undergoing adjuvant chemotherapy. However, evidence remains largely non-randomized, and clinical risk factors continue to guide contemporary practice. To note, the impact of third-line chemotherapy and boost delivery on late toxicity warrants further investigation, particularly in light of several reports evaluating toxicity profiles in hypofractionated RT [68,69]. The omission of boost has not been addressed in complete responders treated with breast-conserving surgery after neoadjuvant pembrolizumab in high-risk early breast cancer, whereas its toxicity has been shown in several reports [70]. Therefore, this subject requires further investigation given the multiplicity of factors involved. While younger age has been confirmed as a fixed parameter to deliver the boost, surgical margins still remains a dependent variable by its status. Although positive margins clearly confer a higher local recurrence risk, the evidence supporting a high dose escalation is limited and accompanied by significantly greater late normal tissue toxicity. Studies are summarized in Table 3. Current guidelines do not support re-excision in case of close margins; all the available evidence suggest that margin width alone should not dictate boost use; a tailored dose escalation to width margins also does not seem to add a certain benefit (Table 4). Radiotherapy delivery has evolved significantly over the last three decades and hypofractionated radiotherapy has become a new standard of care. To date, hypofractionated boost protocols are permitted due to their comparable outcomes with conventional scheduling and are thus endorsed by international guidelines [71]. Further, advanced treatment deliveries like IMRT and VMAT have rendered simultaneous integrated boost (SIB) approaches increasingly attractive, even with conventional fractionation and techniques, by shortening overall treatment time without compromising oncological outcomes (Table 5). This strategy has a favorable impact on quality of life as appeared by PROMs and maintains well the radiobiological advantages aligned with the ASARA principle [72]. While the radiation oncology community is gaining confidence worldwide in utilizing SIB within moderately or escalated hypofractionated WBI (see Table 6 and Table 7), SEB or SIB protocols within ultra-hypofractionated WBI remain under investigation, despite encouraging real-world evidence. A summary is shown in Table 8. The histological features of DCIS remain highly relevant for boost personalization, particularly in the presence of a non-low-risk disease, but again the benefit must be balanced against toxicity and potential quality-of-life impairment as shown by ongoing trials (Table 9). The integration of diverse flap reconstruction techniques into oncoplastic breast-conserving surgery further complicates the delineation of the tumor-bed boost, as substantial data on this approach remain scarce. Nevertheless, the adoption of clip-based contouring guidelines seems to mitigate the intraobserver variability and facilitate adequate volume coverage. The boost topic has not been immune to the evolving challenges characterizing contemporary radiotherapy. Overall, these considerations support a personalized approach to boost prescription based on patient age, tumor biology, surgical margins, systemic therapy, recurrence-risk profile, treatment technique, breast anatomy, and patient preference.

**Table 3:** Clinical evidence on radiation boost dose escalation in positive surgical margins.

Study/Meta-Analysis	Cohort/Patient Population	Boost Regimen & Strategy	Local-Control & Recurrence Outcomes	Late Toxicity & Cosmesis	Survival Outcomes
**Moran et al. (Meta-analysis)**	18 integrated studies evaluating boost in positive margins	Boost utilization analyzed	Risk of local failure remained significantly elevated despite boost (**OR: 2.45**; *p* < 0.001).	Not specified.	Not specified.
**EORTC Subgroup Analysis**	Subgroup of N = 251 patients with positive margins	26 Gy boost vs. 10 Gy boost	Numerically lower but non-significant 10-year IBTR: **10.8% (26 Gy) vs. 17.5% (10 Gy)** (*p* > 0.1).	Moderate-to-severe fibrosis **significantly increased from 24.0% to 54.3%** (*p* < 0.0001).	Dose escalation did not translate into a survival benefit (HR: 0.97; 95% CI: 0.59–1.5; *p* > 0.1).
**Budapest Trial Subgroup**	Subgroup with positive or close margins (9.7% in no-boost; 6.7% in boost arm)	High-dose-rate (**HDR**) brachytherapy boost	**5-year LR**: Reduced from 50.8% to 8.3%. **30-year LRFS**: **77.0% (boost) vs. 20.5% (no boost)**. No difference found in negative margins.	High incidence of **grade 3–4 fat necrosis** in the HDR brachytherapy group.	Clinical benefit offset by late toxicity; no overall survival benefit mentioned.
**Lee et al. (Retrospective)**	Subgroup of N = 55 patients with positive margins out of N = 550	High-dose boost (**15 Gy in 5 fractions** post-50 Gy WBI)	**Crude LR**: 7.3% (positive margins) vs. 2.4% (negative margins); *p* = 0.062. **Age ≤ 40**: Significant reduction in 5-year LRFS vs. negative margins (**89.16% vs. 97.57%**; *p* = 0.005).	Not specified.	**Age ≥ 60**: No significant difference in 5-year LRFS between margin groups (100.00% vs. 94.38%; *p* = 0.426).
**Young Boost Trial**	Randomized trial cohort at 10-year follow-up	26 Gy boost vs. 16 Gy boost	Local relapse rate was **2.8% in the 26 Gy group vs. 4.4% in the 16 Gy group**.	Cumulative incidence of severe fibrosis nearly doubled: **48.0% (26 Gy) vs. 27.0% (16 Gy)**. independent of modality/volume.	Worsened overall cosmetic outcomes due to dose-dependent late toxicity.

**Table 4:** Impact of close margins (≤2 mm) and tailored boost strategies on outcomes.

Study/Guideline	Study Design & Population	Definitional Criteria	Local Recurrence (LR)/IBTR Findings	Key Clinical Conclusions
**Moran et al. (Meta-analysis)**	19 studies (N = 13,081 patients); median follow-up: 8.7 years	Margin widths evaluated at 1, 2, and 5 mm	No statistically significant relationship between specific margin widths and IBTR (*p* = 0.58).	Increasing negative-margin width does not decrease IBTR, even after adjusting for boost or re-excision.
**Shah et al. (Systematic Review)**	38 studies (N = 54,502 patients) treated between 1968 and 2010	Various negative-margin width cohorts	Absolute LR rates decreased over time across all cohorts; maximum difference between groups was **<1%** in recent eras.	Relative rates of LR between different margin width groups remained stable over time despite absolute declines.
**Houssami et al. (Meta-analysis)**	33 studies (N = 28,162 patients; 1506 LR events)	Positive vs. Close vs. Negative margins	Positive vs. Negative: **OR 2.44** (*p* < 0.001). Close vs. Negative: **OR 1.74** (*p* < 0.001). LR was **not associated with margin distance** (*p* = 0.90).	Odds of LR are strongly driven by categorical margin status (positive/close vs. negative) rather than exact numeric distance.
**Vrieling et al. (EORTC Reanalysis)**	EORTC boost trial cohort; 18.2-year follow-up	Comparison of ≤2 mm, 3–4 mm, and ≥5 mm negative margins	Patients across all three margin distance cohorts achieved an **identical LR rate of 10.0%** (*p* = 0.63).	The local-control benefit of the radiation boost is equivalent regardless of the negative-margin width.
**Cho et al.**	N = 297 patients (2000–2012); 18.2% close margins (<1 mm)	Close margins defined as **<1 mm**	Higher radiation doses (**>66 Gy EQD2**) significantly improved local control in the <1 mm cohort.	Aggressive dose escalation may salvage local control in cases with extremely narrow (<1 mm) margins.
**Livi et al.**	Retrospective cohort; median follow-up: 5.2 years; 41 LR events (2%)	**Tailored boost**: 10 Gy (>5 mm), 16 Gy (2–5 mm), 20 Gy (<2 mm)	LR rates: **2.3% (<2 mm), 2.6% (2–5 mm), 1.8% (>5 mm)**. Final margin status did not impact LRFS (*p* = 0.46).	Margin-tailored dose escalation does not modulate LRFS. High nuclear grade, triple-negative status, and T-stage remain the only independent predictors.

*Abbreviations* *: **CI**, confidence interval; **EQD2**, equivalent dose in 2 Gy fractions; **HR**, hazard ratio; **IBTR**, ipsilateral breast tumor recurrence; **LR**, local recurrence; **LRFS**, local recurrence-free survival; **OR**, odds ratio; **WBI**, whole-breast irradiation.*

**Table 5:** Clinical evidence on conventional fractionation and concomitant/simultaneous integrated boost (SIB) regimens.

Trial/Study	Cohort & Study Design	Radiotherapy Regimens & Techniques	Local-Control (LC)/Recurrence Outcomes	Toxicity & Cosmetic Outcomes	Clinical Implications
**Historical SIB Cohorts (since 2005)**	Retrospective/prospective schedules	28 fx: 1.81 Gy (breast) + 0.49 Gy extra (boost) or 21 fx: 2.17 Gy (breast) + 0.49 Gy extra (boost).	Actuarial 5-year LC rate of **98.9%**, effectively minimizing ipsilateral recurrences.	Favorable profile with minimized adverse effects.	Demonstrated early feasibility of integrating SIB within conventional fractionation schemas.
**IMRT-MC2 Trial**	Phase III non-inferiority trial	**SIB arm**: WBI 50.4 Gy + SIB to 64.4 Gy in 28 fx (IMRT). **SEB arm**: WBI 50.4 Gy in 28 fx + 16 Gy sequential boost to 66.4 Gy in 8 fx (3D-CRT).	Median follow-up (5.1 y): 2-year LC was **non-inferior** in the SIB arm (**99.6% vs. 99.6%**; *p* = 0.487).	Non-inferior cosmetic outcomes for IMRT-SIB vs. 3D-SEB at 6 months (9.1% vs. 9.1%) and 2 years (10.4% vs. 9.8%).	First Phase III trial validating the non-inferiority of conventional SIB via IMRT compared to sequential 3D-CRT boost.

**Table 6:** Moderate hypofractionation schedules and dose-escalation strategies (15–16 fractions).

Trial/Regimen	Cohort & Design	Radiotherapy Regimens & Techniques	Local Recurrence (LR)/IBTR Outcomes	Toxicity & Patient-Reported Outcomes (PROMs)	Cosmetic Outcomes
**Franceschini Regimen**	Prospective/Phase II trial framework	40 Gy in 15 fractions to the whole breast + **48 Gy SIB**.	Median follow-up (6 y): Local recurrence risk was exceptionally low at **1.1%**.	Favorable acute and late toxicity profile.	**99.0%** of cases achieved an excellent or good cosmetic outcome.
**16-Fraction SIB Regimen**	Clinical cohort study	40 Gy in 16 fractions to the whole breast + **48 Gy SIB**.	5-year follow-up: Documented a **1.0%** local recurrence rate.	No toxicity reported in 64.0% of cases; low incidence of Grade 3 telangiectasia and tissue edema.	Acceptable aesthetic outcomes with controlled late effects.
**Van Hulle et al.**	Randomized controlled trial (N = 150, prone position)	**SEB**: 40.05 Gy in 15 fx + sequential boost. **SIB**: 46.8 Gy (negative margins) or 49.95 Gy (positive margins).	Shorter treatment duration without compromising efficacy.	2-year data (LENT SOMA scale) showed **no Grade 3 toxicity** or severe late adverse events.	Objective software evaluation (BCCT.core) confirmed no significant cosmetic deterioration between arms.
**RTOG 1005**	Phase III randomized non-inferiority trial (N ≈ 2500 high-risk Stage I–II)	**Arm I (Standard)**: 50 Gy/25 fx or 42.7 Gy/16 fx + sequential boost (12 Gy/6 fx or 14 Gy/7 fx). **Arm II (Hypofx)**: 40 Gy/15 fx + concomitant boost (8 Gy/15 fx; 0.53 Gy/day).	**5-year LR**: 2.0% (Arm I) vs. 1.9% (Arm II). **7-year LR**: 2.2% (Arm I) vs. 2.6% (Arm II). Statistical non-inferiority fully met (***p***** = 0.039**).	Median follow-up (7.3 y): No statistically significant differences in adverse events or overall survival.	Clinical cosmetic assessment at 3 years showed “excellent/good” results in **86.0% (Arm I) vs. 84.0% (Arm II)** (*p* = 0.61).
**Hi-RISE**	Ongoing Phase III non-inferiority trial (Target N = 2904 early breast cancer)	**HFRTsib**: 40 Gy in 15 fx + 48 Gy SIB. **Control**: Conventional fractionation + 10 Gy sequential boost in 5 fx.	Final efficacy endpoints pending; evaluating definitive local-control equivalence.	Safety profile and long-term toxicity parameters under active evaluation.	Cosmetic non-inferiority to be determined upon study completion.
**HYPOSIB**	Ongoing Phase III non-inferiority trial (Target N = 2310 early breast cancer)	**HFRTsib**: 40 Gy in 15 fx + 48 Gy SIB. **Control**: Conventional fractionation + 16 Gy sequential boost in 8 fx.	Final efficacy endpoints pending; evaluating definitive local-control equivalence.	Safety profile and long-term toxicity parameters under active evaluation.	Cosmetic non-inferiority to be determined upon study completion.

**Table 7:** Ongoing dose-escalated and accelerated hypofractionation.

Trial Name/Endpoint	Cohort Size & Follow-up	Treatment Arms & Target Volumes	Local-Control & Recurrence Rates	Late Toxicity Profile	Cosmetic & Acceptance Parameters
**IMPORT HIGH ** *(10-Year Update—ESTRO 2026)*	Long-term update analysis	Same as above (Standard vs. Arm A vs. Arm B).	**10-year KM IBTR**: 3.5% (Standard) vs. 3.7% (Arm A) vs. 5.5% (Arm B). Absolute LR difference: 0.1% (Arm A) vs. 2.2% (Arm B). Cancer-related events: 0.4% (Arm A) vs. 2.5% (Arm B).	Validated the long-term safety and feasibility of the hypofractionated **48 Gy SIB in 15 fractions** using VMAT/IMRT.	Absolute difference in overall survival (OS) at 10 years was minimal: −0.5% in Arm A vs. 1.5% in Arm B.
**NOVEMBER**	Prospective non-inferiority trial (N = 103 patients)	**Ultra-accelerated**: 34.2 Gy in 9 fractions + **39.6 Gy SIB in 9 fractions**.	Median follow-up (51 months): **Zero local recurrences** recorded; only 1 axillary/distant recurrence.	Equivalent and low toxicity at 2 years. **No late toxicity ≥ Grade 3 **. Only 4 patients experienced Grade 2 toxicity.	Cosmetic results at 24 months: **68.0% excellent/good** and 32.0% fair/poor.

*Abbreviations* *: **APBI**, accelerated partial breast irradiation; **ARR**, absolute risk reduction; **3D-CRT**, three-dimensional conformal radiotherapy; **CTV**, clinical target volume; **fx**, fractions; **HFRTsib**, hypofractionated radiotherapy with simultaneous integrated boost; **IBTR**, ipsilateral breast tumor recurrence; **IMRT**, intensity-modulated radiotherapy; **KM**, Kaplan–Meier; **LC**, local control; **LR**, local recurrence; **OS**, overall survival; **PROMs**, patient-reported outcome measures; **PTV**, planning target volume; **SEB**, sequential electron boost; **SIB**, simultaneous integrated boost; **VMAT**, volumetric modulated arc therapy; **WBI**, whole-breast irradiation.*

**Table 8:** Clinical evidence on ongoing study on ultra-hypofractionated radiotherapy and integrated/sequential boost strategies (5-fraction regimens).

Trial/Study	Cohort & Design	Radiotherapy Regimens & Target Volumes	Local-Control (LC) & Survival Outcomes	Acute & Late Toxicity Profiles	Clinical Status & Implications
**UK FAST-Forward Boost** (Ref [52])	Large-scale Phase III randomized non-inferiority trial (Target N > 4000; Active Recruitment)	**Control**: 48 Gy SIB in 15 fractions (3 weeks). **Experimental arms**: 31 Gy and 30 Gy SIB in **5 fractions (1 week)**.	Long-term efficacy and local-control endpoints pending study maturation.	Aiming to establish late toxicity and cosmetic non-inferiority compared to the 3-week standard.	Designed to test if a 1-week ultra-hypofractionated SIB is non-inferior to the 3-week IMPORT High standard.
**HYPORT Adjuvant Study** (Ref [53])	Randomized trial; Interim analysis	**Control SIB**: 40 Gy in 15 fractions + 8 Gy SIB. **Ultra-HYPOSIB**: 26 Gy in 5 fractions + **6 Gy SIB**.	Efficacy data pending further follow-up.	Grade 3 acute dermatitis occurred in only 3/271 patients (none in the SIB arm). Minimal incidence of severe acute toxicity, with **no significant difference** between SIB schedules.	Interim analysis confirms the acute safety of an ultra-hypofractionated 5-fraction SIB regimen.
**MC1635 Trial** (Ref [54])	Randomized controlled trial (Awaiting definitive results)	**Control SIB**: 40 Gy in 15 fractions + 8 Gy SIB. **Ultra-HYPOSIB**: 26 Gy in 5 fractions + **4 Gy SIB**.	Efficacy, local control, and survival analysis ongoing.	Physician- and patient-reported outcomes (PROMs) under active evaluation.	Investigating the therapeutic window and cosmetic outcomes of a highly de-escalated 4-Gy ultra-hypofractionated SIB.

*Abbreviations* *: CI, confidence interval; fx, fractions; LRFS, local recurrence-free survival; MV, megavoltage; OS, overall survival; SEB, sequential electron boost; SIB, simultaneous integrated boost; WBI, whole-breast irradiation.*

**Table 9:** Clinical efficacy and late toxicity of boost irradiation in ductal carcinoma in situ (DCIS).

Trial/Study	Design & Patient Population	Non-Low-Risk Definitional Criteria	Local-Control & Recurrence Outcomes	Late Toxicity, Quality of Life (QoL) & Cosmesis	Survival Outcomes
**BIG 3-07/TROG 07.01**	Phase III randomized, factorial, 4-arm design. N = 1608 patients with non-low-risk DCIS. Compares boost vs. no boost combined with conventional vs. hypofractionated WBI.	Age < 50 years, symptomatic presentation, palpable mass, lesion size ≥ 15 mm, multifocality, intermediate/high nuclear grade, central necrosis, comedo histology, or radial surgical margin < 10 mm.	**10-year RFS**: 93.2% (boost) vs. 86.0% (no boost). **10-year freedom from disease recurrence**: **86.5% vs. 79.4%** (HR: 0.67; 95% CI: 0.52–0.86; *p* = 0.002). **WBI Fractionation**: Hypofractionation was **non-inferior** to conventional WBI (10-year freedom from local recurrence: 88.6% vs. 89.5%; *p* = 0.82).	Explicit late toxicity profile detailed in parallel updates; the de-escalation of WBI via hypofractionation did not compromise safety.	**10-year OS**: 95.7% (boost) vs. 93.9% (no boost). No statistically significant difference demonstrated (**HR: 0.73**; 95% CI: 0.47–1.14; *p* = 0.17).
**Australian-SWG Study**	Clinical cohort study with a median follow-up of 6.6 years.	Enrolled patients with diagnosed DCIS.	**Local Recurrence (LR) Rate**: Significantly reduced from **7.3% (no boost) to 2.9% (boost)** (*p* < 0.001).	Significant reduction in local failure was achieved at the expense of an **increased incidence of adverse effects**.	Not reported/No significant overall survival variation.
**French BONBIS Trial**	Phase III randomized trial. N = 2000 patients. Evaluates a **16 Gy boost in 8 fractions** vs. no boost.	Enrolled patients with diagnosed DCIS.	Primary efficacy endpoints met; long-term local-control benefit confirmed.	**Grade ≥ 2 Breast Subcutaneous Fibrosis (BSCF)**: Significantly increased from **2.8% to 7.0%** (*p* < 0.001). **BSCF Risk Factors**: Strictly correlated with boost delivery (**OR: 2.6;** 95% CI: 1.62–4.3) and a breast **CTV ≥ 500 cm^3^** (OR: 1.6; 95% CI: 1.01–2.51). **QoL (QLQ-C30/QLQ-B23)**: Higher frequency of **persistent breast symptoms **(*p* = 0.001) and an early detriment in **body image** (HR: 1.19; 95% CI: 1.00–1.41).	

## 11. Future Directions

The role of tumor-bed boost irradiation remains an active field of investigation. Future studies should focus on identifying patients in whom boost omission is truly safe in the setting of modern systemic therapy, clarifying the role of boost escalation in positive-margin disease, and defining the long-term safety of ultra-hypofractionated SIB schedules. Moreover, the impact of new oncoplastic surgery techniques within hypofractionated boost schedules needs to be clarified. More mature and definitive data are awaited from ongoing clinical trials. Prospective or randomized trials are also needed to integrate the role of systemic therapy in boost omission, clinical and probably also studies integrating pathological, and molecular risk factors into individualized decision-making models are required. In addition, future research should give greater emphasis to patient-reported outcomes, cosmetic results, and quality of life, which are highly relevant when absolute differences in local recurrence become progressively smaller.

## 12. Conclusions

Tumor-bed boost irradiation remains a valuable component of adjuvant whole-breast radiotherapy for selected patients with early-stage breast cancer. Historical randomized trials and subsequent meta-analyses have consistently shown that a boost improves local control, particularly in younger patients and those at higher baseline risk of recurrence. However, this benefit has not been associated with a consistent survival advantage and is accompanied by increased late toxicity.

In the era of modern systemic therapy, surgery and advanced radiotherapy delivery, routine boost administration to all patients is increasingly difficult to justify. Instead, treatment decisions should be individualized according to the predicted absolute benefit in local control, surgical margin status, tumor biology, fractionation schedule, target volume, and patient preferences. Ongoing clinical trials will be crucial in further refining the role of boost irradiation and establishing more personalized treatment strategies.

## Figures and Tables

**Table 1:** Overview of randomized controlled trials on breast radiotherapy boost.

Trial Name	Sample Size (*N*)	Median Follow-Up	Local-Control Outcomes	Toxicity & Cosmetic Outcomes	Survival Outcomes
**Lyon ** **(electrons)**	1024	3.3 years	Significant reduction in 5-year local recurrence rate.	Increased incidence of grade 1–2 telangiectasia.	Not reported/No significant difference.
**Budapest ** **(electrons) ** **(HDR brachytherapy)**	207	30 years	Significant reduction in crude local recurrence; equivalent efficacy between electrons and HDR brachytherapy.	Significantly higher rate of adverse side effects in the boost arm.	30-year CSS significantly improved (**62.0% vs. 51.5%**, (*p* = 0.0282)). Trend toward improved DFS (45.0% vs. 38.9%, (*p* = 0.0806)). No OS benefit (33.0% vs. 27.9%, (*p* = 0.1182)). Greatest benefit in patients aged (le) 40.
**Australian ** **(electrons)**	688	8.5 years	Local recurrence was **2.0% in control vs. 4.4% in boost arm**; reducing whole-breast dose (45 Gy vs. 50 Gy) negates boost benefit.	5-year cosmetic outcomes (via breast retraction assessment) suggest whole-breast dose reduction offsets potential boost advantages.	Not reported/No significant difference.
**Nice ** **(photons with ** **Co80 unit)**	664	6 years	No statistically significant difference in local recurrence (**4.3% in Group B [boost] vs. 6.8% in Group A [no boost]**).	Not reported.	Not reported/No significant difference.
**EORTC ** **(photons, electrons, HDR-brachy)**	2657 *	10 to 20 years	Significant improvement in local control across all ages. 10-year cumulative local recurrence: **6.2% (boost) vs. 10.2% (no boost)**. Pronounced 10-year ARR in patients aged (le) 40 (**13.5% vs. 23.9%**).	Significantly higher rate of 10-year severe fibrosis in the boost arm (**4.4% vs. 1.6%**).	No survival benefit demonstrated at 10 years. 20-year OS showed no statistical difference (**59.7% boost vs. 61.1% no boost**; HR: 1.05, 99% CI: 0.92–1.19, (*p* = 0.323)).

*Abbreviations* : **ARR**, absolute risk reduction; **CI**, confidence interval; **CSS**, cancer-specific survival; **DFS**, disease-free survival; **HDR**, high-dose-rate; **HR**, hazard ratio; **OS**, overall survival; **WBI**, whole-breast irradiation. * Note: (N = 2657) refers to the randomized cohort with macroscopically excised tumors within the broader 5569-patient trial framework.

**Table 2:** Evidence synthesis from systematic reviews and consensus guidelines on breast boost radiotherapy.

Study/Consensus	Design/Population	Primary Clinical Findings	Toxicity & Cosmetic Outcomes	Therapeutic Recommendations
**Kindts et al. (Cochrane Review) [5]**	Meta-analysis of randomized controlled trials (N = 8315)	Significant improvement in local control (HR: 0.64; 95% CI: 0.55–0.75; (*p* < 0.00001)). Local control remained superior in women aged >40 years (HR: 0.65; 95% CI: 0.53–0.81; *p* < 0.0001). No differences observed in **DFS** or **OS**.	Panel-assessed cosmetic outcomes were significantly worse in the boost arm. Physician-reported cosmetic results showed no significant differences.	Tumor-bed boost irradiation is validated as a standard option to enhance local control across various age groups.
**Kayali et al. (Systematic Review) [6]**	Comprehensive evaluation of 7 clinical studies	6 out of 7 studies reported significant local-control benefit (HR range: 0.34–0.73). The largest absolute local-control benefit was observed in younger cohorts. No survival advantage (**OS**) was demonstrated in any included study.	Late fibrosis was consistently more frequent in the boost group. Cumulative incidence of severe fibrosis was significantly higher with boost therapy across all age cohorts > 40 years (*p* < 0.001).	The clinical utility of the boost lies primarily in local control rather than survival extension. Prescriptions must be individualized based on recurrence risk vs. late toxicity tolerance.
**Assisi Think Tank Consensus (ATTC) 2024 [7]**	Expert consensus and clinical guideline framework	*Clinical threshold set at 10-year absolute risk reduction (ARR) for local recurrence.*	Implicit consideration of late toxicity trade-offs within the risk-stratification model.	**Omission of boost** is contemplated if estimated 10-year ARR is <3%. **Shared decision-making** is mandated if estimated 10-year ARR is **>3%.**

*Abbreviations: **ARR**, absolute risk reduction; **CI**, confidence interval; **DFS**, disease-free survival; **HR**, hazard ratio; **OS**, overall survival* [1,2].

## Data Availability

Data are available in the reference notes.

## References

[1] Holland R., Connolly J.L., Gelman R., Mravunac M., Hendriks J.H., Verbeek A.L., Schnitt S.J., Silver B., Boyages J., Harris J.R. (1990). The presence of an extensive intraductal component following a limited excision correlates with prominent residual disease in the remainder of the breast. J. Clin. Oncol..

[2] Yiu C.C.P., Loo W.T.Y., Lam C.K., Chow L.W.C. (2009). Presence of extensive intraductal component in patients undergoing breast conservative surgery predicts presence of residual disease in subsequent completion mastectomy. Chin. Med. J..

[3] Bartelink H., Horiot J.C., Poortmans P.M., Struikmans H., Van den Bogaert W., Fourquet A., Jager J.J., Hoogenraad W.J., Oei S.B., Wárlám-Rodenhuis C.C. (2007). Impact of a higher radiation dose on local control and survival in breast-conserving therapy of early breast cancer: 10-year results of the randomized boost versus no boost EORTC 22881-10882 trial. J. Clin. Oncol..

[4] Bartelink H., Maingon P., Poortmans P., Weltens C., Fourquet A., Jager J., Schinagl D., Oei B., Rodenhuis C., Horiot J.-C. (2015). Whole-breast irradiation with or without a boost for patients treated with breast-conserving surgery for early breast cancer: 20-year follow-up of a randomised phase 3 trial. Lancet Oncol..

[5] Kindts I., Laenen A., Depuydt T., Weltens C. (2017). Tumour bed boost radiotherapy for women after breast-conserving surgery. Cochrane Database Syst. Rev..

[6] Kayali M., Jaoude J.A., Ramia P., Assi H., Geara F., Poortmans P., Zeidan Y.H. (2021). Post-lumpectomy radiation therapy boost in breast cancer patients: Evidence revisited. Ecancermedicalscience.

[7] Arenas M., Bölükbaşı Y., Boersma L.J., Offersen B., Kouloulias V., Palumbo I., Trigo L., Lozza L., Marazzi F., Trovo M. (2025). The 2024 Assisi think tank on breast cancer: Focus on the use of a tumour bed boost after breast conserving therapy. Breast.

[8] Fisher B., Gunduz N., Coyle J., Rudock C., Saffer E. (1989). Presence of a growth-stimulating factor in serum following primary tumor removal in mice. Cancer Res..

[9] Knutson K.L., Lu H., Stone B., Reiman J.M., Behrens M.D., Prosperi C.M., A Gad E., Smorlesi A., Disis M.L. (2006). Immunoediting of cancers may lead to epithelial to mesenchymal transition. J. Immunol..

[10] Nuyten D.S., Kreike B., Hart A.A., Chi J.-T.A., Sneddon J.B., Wessels L.F., Peterse H.J., Bartelink H., O Brown P., Chang H.Y. (2006). Predicting a local recurrence after breast-conserving therapy by gene expression profiling. Breast Cancer Res..

[11] Belletti B., Vaidya J.S., D’Andrea S., Entschladen F., Roncadin M., Lovat F., Berton S., Perin T., Candiani E., Reccanello S. (2008). Targeted intraoperative radiotherapy impairs the stimulation of breast cancer cell proliferation and invasion caused by surgical wounding. Clin. Cancer Res..

[12] Chakraborty M., Abrams S.I., Camphausen K., Liu K., Scott T., Coleman C.N., Hodge J.W. (2003). Irradiation of tumor cells up-regulates Fas and enhances CTL lytic activity and CTL adoptive immunotherapy. J. Immunol..

[13] Formenti S.C., Demaria S. (2008). Local control by radiotherapy: Is that all there is?. Breast Cancer Res..

[14] Romestaing P., Lehingue Y., Carrie C., Coquard R., Montbarbon X., Ardiet J.M., Mamelle N., Gérard J.P. (1997). Role of a 10-Gy boost in the conservative treatment of early breast cancer: Results of a randomized clinical trial in Lyon, France. J. Clin. Oncol..

[15] Polgár C., Fodor J., Orosz Z., Major T., Takácsi-Nagy Z., Mangel L.C., Sulyok Z., Somogyi A., Kásler M., Németh G. (2002). Electron and high-dose-rate brachytherapy boost in the conservative treatment of stage I–II breast cancer: First results of the randomized Budapest boost trial. Strahlenther. Onkol..

[16] Polgár C., Takacsi-Nagi Z., Major T., Fodor J. (2026). Electron and brachytherapy boost after breast-conserving surgery and whole breast irradiation: 30-year results of the randomized for Radiotherapy and Oncology. Radiother. Oncol..

[17] Hau E., Browne L., Capp A., Delaney G.P., Fox C., Kearsley J.H., Millar E., Nasser E.H., Papadatos G., Graham P.H. (2013). The impact of breast cosmetic and functional outcomes on quality of life: Long-term results from the St. George and Wollongong randomized breast boost trial. Breast Cancer Res. Treat..

[18] Early Breast Cancer Trialists’ Collaborative Group (EBCTCG) (2005). Effects of chemotherapy and hormonal therapy for early breast cancer on recurrence and 15-year survival: An overview of the randomised trials. Lancet.

[19] Braybrooke J., Bradley R., Hills R.K., Peto R., Kerr A., Pan H., Clarke M., Dodwell D., McGale P., Taylor C. (2025). Extending the duration of endocrine treatment for early breast cancer: Patient-level meta-analysis of 12 randomised trials of aromatase inhibitors in 22,031 postmenopausal women already treated with at least 5 years of endocrine therapy. Lancet.

[20] Kayali M., Jaoude J.A., Tamim H., Makki M., Tfaily A., El Saghir N., Assi H., De Azambuja E., Poortmans P., Geara F. (2020). De-intensifying radiation therapy in HER-2 positive breast cancer: To boost or not to boost?. Int. J. Radiat. Oncol. Biol. Phys..

[21] Cao L., Cai G., Xu F., Yang Z.Z., Yu X.L., Ma J.L., Zhang Q., Wu J., Guo X.M., Chen J.Y. (2016). Trastuzumab improves locoregional control in HER2-positive breast cancer patients following adjuvant radiotherapy. Medicine.

[22] Verreck E.E.F., Heemsbergen W.D., van Dalen T., Windhorst S., de Munck L., van der Leij F., Boersma L.J., Froklage F.E. (2026). Is a boost to the tumour bed still indicated after breast-conserving surgery and whole-breast radiotherapy in the era of modern systemic therapy?. Radiother. Oncol..

[23] Wazer D.E., Schmidt-Ullrich R.K., Ruthazer R., Schmid C.H., Graham R., Safaii H., Rothschild J., McGrath J., Erban J.K. (1998). Factors determining outcome for breast conserving irradiation with margin-directed dose escalation to the tumor bed. Int. J. Radiat. Oncol. Biol. Phys..

[24] Moran M.S., Schnitt S.J., Giuliano A.E., Harris J.R., Khan S.A., Horton J., Klimberg S., Chavez-MacGregor M., Freedman G., Houssami N. (2014). Society of Surgical Oncology–American Society for Radiation Oncology consensus guideline on margins for breast-conserving surgery with whole-breast irradiation in stages I and II invasive breast cancer. Int. J. Radiat. Oncol. Biol. Phys..

[25] Poortmans P.M., Collette L., Horiot J.C., Van den Bogaert W., Fourquet A., Kuten A., Noordijk E.M., Hoogenraad W., Mirimanoff R.-O., Pierart M. (2009). Impact of the boost dose of 10 Gy versus 26 Gy in patients with early stage breast cancer after a microscopically incomplete lumpectomy: 10-year results of the randomised EORTC boost trial. Radiother. Oncol..

[26] Kim H., Kim T.G., Park B., Kim J.H., Jun S.-Y., Lee J.H., Choi H.J., Jung C.S., Bang Y.J., Lee H.W. (2023). Effect of high-dose radiation therapy on positive margins after breast-conserving surgery for invasive breast cancer. Breast.

[27] Bosma S., van Werkhoven E., Bartelink H., Fourquet A., Hurkmans C., Maduro J., Rutgers E., Scheijmans L., Schinagl D., Stam M. (2024). Young boost randomized phase III trial of high vs low boost radiation in young breast cancer patients: 10-year results. Eur. J. Cancer.

[28] Ceilley E., Jagsi R., Goldberg S., Grignon L., Kachnic L., Powell S., Taghian A. (2005). Radiotherapy for invasive breast cancer in North America and Europe: Results of a survey. Int. J. Radiat. Oncol. Biol. Phys..

[29] Nguyen K., Mackenzie P., Allen A., Dreosti M., Morgia M., Zissiadis Y., Lamoury G., Windsor A. (2017). Australian and New Zealand patterns of practice survey on breast radiotherapy. J. Med. Imaging Radiat. Oncol..

[30] Tyler S., Truong P.T., Lesperance M., Nichol A., Baliski C., Warburton R., Tyldesley S. (2018). Close margins less than 2 mm are not associated with higher risks of 10-year local recurrence and breast cancer mortality compared with negative margins in women treated with breast-conserving therapy. Int. J. Radiat. Oncol. Biol. Phys..

[31] Kaufmann M., Morrow M., von Minckwitz G., Harris J.R. (2010). Locoregional treatment of primary breast cancer: Consensus recommendations from an international expert panel. Cancer.

[32] Shah C., Hobbs B.P., Vicini F., Al-Hilli Z., Manyam B.V., Verma V., Jia X., Goldstein N., Recht A. (2020). The diminishing impact of margin definitions and width on local recurrence rates following breast-conserving therapy for early-stage invasive cancer: A meta-analysis. Ann. Surg. Oncol..

[33] Houssami N., Macaskill P., Marinovich M.L., Morrow M. (2014). The association of surgical margins and local recurrence in women with early-stage invasive breast cancer treated with breast-conserving therapy: A meta-analysis. Ann. Surg. Oncol..

[34] Vrieling C., van Werkhoven E., Maingon P., Poortmans P., Weltens C., Fourquet A., Schinagl D., Oei B., Rodenhuis C.C., Horiot J.-C. (2017). Prognostic factors for local control in breast cancer after long-term follow-up in the EORTC boost vs no boost trial: A randomized clinical trial. JAMA Oncol..

[35] Cho W.K., Choi D.H., Park W., Kim H., Cha H. (2019). Is higher dose radiation necessary for positive resection margin after breast-conserving surgery for breast cancer?. Breast.

[36] Livi L., Meattini I., Franceschini D., Saieva C., Meacci F., Marrazzo L., Gerlain E., Desideri I., Scotti V., Nori J. (2013). Radiotherapy boost dose-escalation for invasive breast cancer after breast-conserving surgery: 2093 patients treated with a prospective margin-directed policy. Radiother. Oncol..

[37] Bantema-Joppe E.J., Vredeveld E.J., de Bock G.H., Busz D.M., Iersel M.W.-V., Dolsma W.V., van der Laan H.P., Langendijk J.A., Maduro J.H. (2013). Five year outcomes of hypofractionated simultaneous integrated boost irradiation in breast conserving therapy; patterns of recurrence. Radiother. Oncol..

[38] Hörner-Rieber J., Forster T., Hommertgen A., Haefner M.F., Arians N., König L., Harrabi S.B., Schlampp I., Weykamp F., Lischalk J.W. (2021). Intensity modulated radiation therapy (IMRT) with simultaneously integrated boost shortens treatment time and is noninferior to conventional radiation therapy followed by sequential boost in adjuvant breast cancer treatment: Results of a large randomized phase III trial (IMRT-MC2 trial). Int. J. Radiat. Oncol..

[39] Vicini F.A., Winter K., Freedman G.M., Arthur D.W., Rosenstein B.S., Bentzen S.M., Li X.A., Halyard M.Y., Woodward W.A., Bleicher R.J. (2026). Concurrent versus sequential radiation dose escalation to the surgical cavity for conservative treatment of high-risk early breast cancer: NRG/RTOG 1005 phase III trial. J. Clin. Oncol..

[40] Franceschini D., Fogliata A., Spoto R., Dominici L., Faro L.L., Franzese C., Comito T., Lobefalo F., Reggiori G., Cozzi L. (2021). Long term results of a phase II trial of hypofractionated adjuvant radiotherapy for early-stage breast cancer with volumetric modulated arc therapy and simultaneous integrated boost. Radiother. Oncol..

[41] Pfaffendorf C., Vonthein R., Krockenberger-Ziegler K., Dellas K., Schreiber A., Uhlemann D., Dinges S., Würschmidt F., Andreas P., Weinstrauch E. (2022). Hypofractionation with simultaneous integrated boost after breast-conserving surgery: Long term results of two phase-II trials. Breast.

[42] Van Hulle H., Desaunois E., Vakaet V., Paelinck L., Schoepen M., Post G., Van Greveling A., Speleers B., Mareel M., De Neve W. (2021). Two-year toxicity of simultaneous integrated boost in hypofractionated prone breast cancer irradiation: Comparison with sequential boost in a randomized trial. Radiother. Oncol..

[43] Coles C.E., Haviland J.S., Kirby A.M., Griffin C.L., Sydenham M.A., Titley J.C., Bhattacharya I., Brunt A.M., Chan H.Y.C., Donovan E.M. (2023). Dose-escalated simultaneous integrated boost radiotherapy in early breast cancer (IMPORT HIGH): A multicentre, phase 3, non-inferiority, open-label, randomised controlled trial. Lancet.

[44] Krug D., Dellas K., Schreiber A., Boicev A., Zimmer J., Uhlemann D., Laubach R., Weidner N., Dinges S., Hipp M. (2024). Hypofractionated whole-breast irradiation with simultaneous integrated boost for breast cancer: Primary analysis of the HYPOSIB-Trial (ARO 2013-05). Int. J. Radiat. Oncol. Biol. Phys..

[45] Coles C. (2026). Ten-year results of the IMPORT HIGH trial (ISRCTN47437448): Dose escalated simultaneous integrated boost radiotherapy in early breast cancer European Society for Radiotherapy and Oncology. Radiother. Oncol..

[46] Jin K., Luo J., Guo X. (2024). Hypofractionated radiotherapy with simultaneous tumor bed boost (Hi-RISE) in breast cancer patients receiving upfront breast-conserving surgery: Study protocol for a phase III randomized controlled trial. Radiat. Oncol..

[47] Poppe M.M., Boucher K., Gaffney D.K., Brownson K.E., Smith G., Howell J.N., Ticona F.F., Kim J., Burt L., Cannon D. (2025). NOVEMBER, A phase 2 trial of a 9-day course of whole breast radiation therapy with a simultaneous lumpectomy boost for early-stage breast cancer. Int. J. Radiat. Oncol. Biol. Phys..

[48] Chakraborty M.A., Khan A.J., Tadros A.B., White C., Zhang Z., Kim M., Xu A.J., LaPlant Q., O’Brien D.R., Cuaron J.J. (2026). Ultrahypofractionated whole breast radiation therapy using a novel boost regimen for early-stage breast cancer. Pract. Radiat. Oncol..

[49] Zheng S.Y., Huang J.Q., Xie J.R., Gan L., Yu B., Shi Y.G., Jiang J., Zhang J., Dong M.L., Qi W.X. (2026). Early safety of ultra-hypofractionated whole breast irradiation and sequential tumor bed boost for early breast cancer (SHIFT): A multicenter, phase 2 trial. Int. J. Radiat. Oncol. Biol. Phys..

[50] Kirby A.M., Cafferty F.H., Poole K., Anandadas C., Bower L., Sydenham M.A., Fleming H., Somaiah N., Grandon V., Nabi Z. (2025). Testing a 5-fraction simultaneous integrated boost in radiotherapy for breast cancer: The UK FAST-Forward Boost Trial opens to recruitment. Clin. Oncol..

[51] Chakraborty S., Chatterjee S., Backianathan S., Lal P., Gupta S., Ahmed R., Misra S., Solomon P., Balakrishan R., Bhushal S. (2022). HYPORT adjuvant acute toxicity and patient dosimetry quality assurance results: Interim analysis. Radiother. Oncol..

[52] Laughlin B.S., Corbin K.S., Toesca D.A.S., Thorpe C.S., Golafshar M.A., Pockaj B., Cronin P., McGee L.A., Halyard M.Y., Mutter R.W. (2024). Physician and patient reported outcomes of the MC1635 phase 3 trial of ultrahypofractionated versus moderately hypofractionated adjuvant radiation therapy after breast conserving surgery. Int. J. Radiat. Oncol. Biol. Phys..

[53] Meattini I., Marrazzo L., Saieva C., Desideri I., Scotti V., Simontacchi G., Bonomo P., Greto D., Mangoni M., Scoccianti S. (2020). Accelerated partial-breast irradiation compared with whole-breast irradiation for early breast cancer: Long-term results of the randomized phase III APBI-IMRT-Florence trial. J. Clin. Oncol..

[54] Palumbo I., Becchetti A.G., D’Angelo E., DeSantis M.C., Fozza A., Lazzari G., Falivene S., Baldissera A., Ingrosso G., De Felice F. (2026). Ultrahypofractionated whole breast irradiation in early-stage breast cancer: An Italian multicenter observational study. Int. J. Radiat. Oncol. Biol. Phys..

[55] Marinovich M.L., Azizi L., Macaskill P., Irwig L., Morrow M., Solin L.J., Houssami N. (2016). The association of surgical margins and local recurrence in women with ductal carcinoma in situ treated with breast-conserving therapy: A meta-analysis. Ann. Surg. Oncol..

[56] Morrow M., Van Zee K.J., Solin L.J., Houssami N., Chavez-MacGregor M., Harris J.R., Horton J., Hwang S., Johnson P.L., Marinovich M.L. (2016). Society of Surgical Oncology–American Society for Radiation Oncology–American Society of Clinical Oncology consensus guideline on margins for breast-conserving surgery with whole-breast irradiation in ductal carcinoma in situ. Ann. Surg. Oncol..

[57] Chua B.H., Link E.K., Olivotto I.A., Kunkler I., Whelan T., Deseyne P., Gruber G. (2022). Abstract GS2-12: Radiation doses and fractionation schedules in non-low-risk ductal carcinoma in situ in the breast (BIG 3-07/TROG 07.01): Final 10-year analysis of a randomised, factorial, multicentre, open-label, phase 3 study. Lancet.

[58] Bourgier C., Mollevi C., Cowen D., Castan F., Lemanski C., Gourgou S., Rivera S., Labib A., Peignaux K., Le Blanc-Onfroy M. (2026). Late toxicities and quality of life after a radiation boost for breast ductal carcinoma in situ: Boost or no boost in DCIS randomized trial. Int. J. Radiat. Oncol. Biol. Phys..

[59] Schmidt G., Mayo T., von Falkenhausen A., Kiechle M., Müller D. (2025). Local perforator flaps in oncoplastic breast surgery: Clinical applications of ICAP and TDAP flaps in reconstruction and complication management. Geburtshilfe Frauenheilkd..

[60] Garreffa E., Meattini I., Coles C.E., Agrawal A. (2023). Use of tumour bed boost radiotherapy in volume replacement oncoplastic breast surgery: A systematic review. Crit. Rev. Oncol. Hematol..

[61] Yarnold J., Bentzen S.M., Coles C., Haviland J. (2011). Hypofractionated whole breast radiotherapy for women with early breast cancer: Myths and realities. Int. J. Radiat. Oncol. Biol. Phys..

[62] Harrington K.J., Harrison M., Bayle P., Evans K., Dunn P.A., Lambert H.E., Saidan Z., Lynn J., Stewart J.W. (1996). Surgical clips in planning the electron boost in breast cancer: A qualitative and quantitative evaluation. Int. J. Radiat. Oncol. Biol. Phys..

[63] Boersma L.J., Janssen T., Elkhuizen P.H., Poortmans P., van der Sangen M., Scholten A.N., Hanbeukers B., Duppen J.C., Hurkmans C., van Vliet C. (2012). Reducing interobserver variation of boost-CTV delineation in breast conserving radiation therapy using a pre-operative CT and delineation guidelines. Radiother. Oncol..

[64] Coles C.E., Wilson C.B., Cumming J., Benson J.R., Forouhi P., Wilkinson J.S., Jena R., Wishart G.C. (2009). Titanium clip placement to allow accurate tumour bed localisation following breast conserving surgery: Audit on behalf of the IMPORT Trial Management Group. Eur. J. Surg. Oncol..

[65] Coles C.E., Harris E.J., Donovan E.M., Bliss P., Evans P.M., Fairfoul J., Mackenzie C., Rawlings C., Syndikus I., Twyman N. (2011). Evaluation of implanted gold seeds for breast radiotherapy planning and on treatment verification: A feasibility study on behalf of the IMPORT trialists. Radiother. Oncol..

[66] Dzhugashvili M., Veldeman L., Kirby A.M. (2023). The role of the radiation therapy breast boost in the 2020s. Breast.

[67] Schneeweiss A., Chia S., Hickish T., Harvey V., Eniu A., Waldron-Lynch M., Eng-Wong J., Kirk S., Cortés J. (2018). Long-term efficacy analysis of the randomised, phase II TRYPHAENA cardiac safety study: Evaluating pertuzumab and trastuzumab plus standard neoadjuvant anthracycline-containing and anthracycline-free chemotherapy regimens in patients with HER2-positive early breast cancer. Eur. J. Cancer.

[68] Hijal T., Al Hamad A.A., Niazi T., Sultanem K., Bahoric B., Vuong T., Muanza T. (2010). Hypofractionated radiotherapy and adjuvant chemotherapy do not increase radiation-induced dermatitis in breast cancer patients. Curr. Oncol..

[69] Kouloulias V., Zygogianni A., Kypraiou E., Georgakopoulos J., Thrapsanioti Z., Beli I., Mosa E., Psyrri A., Antypas C., Armbilia C. (2014). Adjuvant chemotherapy and acute toxicity in hypofractionated radiotherapy for early breast cancer. World J. Clin. Cases.

[70] Lazzari G., Benevento I., D’Andrea B., Prudente A., Brucoli I., Bianculli A., Tucciariello R., Lerose R., Metallo V., Solazzo A. (2025). The impact of KEYNOTE-522 related immune-related adverse events on concurrent adjuvant whole breast radiotherapy in the real world: A case series: KEYNOTE-522 and adjuvant hypofractionated RT. Arch. Breast Cancer.

[71] Smith B.D., Bellon J.R., Blitzblau R., Freedman G., Haffty B., Hahn C., Halberg F., Hoffman K., Horst K., Moran J. (2018). Radiation therapy for the whole breast: Executive summary of an American Society for Radiation Oncology (ASTRO) evidence-based guideline. Pract. Radiat. Oncol..

[72] Mackillop W.J., Bates J.H.T., O’Sullivan B., Withers H.R. (1996). The effect of delay in treatment on local control by radiotherapy. Int. J. Radiat. Oncol. Biol. Phys..

